# The Hearing–Cognition Relationship Across the Lifespan: Mechanisms, Interventions and Outcomes

**DOI:** 10.3390/audiolres16050141

**Published:** 2026-09-22

**Authors:** Christos Tsilivigkos, Vasileios Papanikos, Polyniki Kastanioudaki, Eleni Bekou, Theodora Skoura, Irida Pano, Francesk Mulita, Vasileios Leivaditis

**Affiliations:** 1 First Department of Otolaryngology, Hippokration General Hospital, National and Kapodistrian University of Athens (NKUA), 15772 Athens, Greece; tsilivig@med.uoa.gr; 2 ENT/Head and Neck Surgery, St George’s Hospital, St George’s University Hospitals NHS Foundation Trust, London SW17 0QT, UK; papanikos.vas@gmail.com; 3 Department of Medicine, School of Health Sciences, University of Ioannina, 45333 Ioannina, Greece; pkastanioudaki@gmail.com; 4 Medical Physics Laboratory, School of Medicine, Democritus University of Thrace, 68100 Alexandroupolis, Greece; bekou1995@yahoo.com; 5 Medical School, Aretaeion Hospital, National and Kapodistrian University of Athens (NKUA), 11528 Athens, Greece; tskoura@yahoo.com; 6 Department of Clinical Subject, University of Medicine Tirana, 1005 Tirana, Albania; 7 General University Hospital of Patras, 26504 Patras, Greece; 8 Westpfalz Klinikum, 67655 Kaiserslautern, Germany; vnleivaditis@gmail.com

**Keywords:** hearing loss, presbycusis, cognitive decline, dementia, mild cognitive impairment, hearing aids, cochlear implants, auditory rehabilitation, cognition, children, young adults, middle-aged adults, lifespan

## Abstract

**Introduction**: Hearing loss and cognitive impairment are among the most common chronic conditions of aging, and a growing body of evidence links the two. The 2020 and 2024 Lancet Commissions identified midlife hearing loss as one of the largest potentially modifiable risk factors for dementia, raising the possibility that auditory rehabilitation could help preserve cognition. **Methods**: This narrative review provides a lifespan perspective by summarizing current evidence on the relationship between hearing loss and cognitive decline, the cognitive domains most affected, and the mechanisms proposed to explain the association, including the cognitive load, common cause, cascade, and overdiagnosis hypotheses, together with their neurobiological correlates. **Results**: We then examine the effect of auditory interventions on cognition in adults, considering hearing aids and cochlear implants separately, and discuss landmark trials such as ACHIEVE, which suggest that cognitive benefits may be greatest in older adults at increased risk of decline. Finally, we address the distinct situation of children, in whom early auditory deprivation may shape the development of working memory and executive function and in whom the timely fitting of hearing devices is critical. **Conclusions**: Overall, the evidence supports hearing loss as a modifiable contributor to cognitive decline and highlights early detection and rehabilitation as promising, though not universally effective, strategies for protecting cognitive health.

## 1. Introduction

People in modern societies tend to live longer, but increased longevity comes at a cost. More than 80% of older adults in the USA live with at least one chronic condition, and approximately half live with two or more; chronic diseases are also the leading cause of death in this age group [1]. Dementia is a chronic, progressive neurodegenerative syndrome. Approximately 50 million people worldwide were living with dementia at the time of the 2018 World Alzheimer Report, a figure projected to rise to about 152 million by 2050 [2]. Hearing loss is likewise an extremely common age-related condition: globally, more than 1.5 billion people experience some degree of hearing loss [3,4], and roughly two-thirds of adults older than 70 years are affected. Because both conditions become more prevalent with age, they frequently coexist in the same individual.

Cognition encompasses a range of functions, including perception, memory (working and long-term), attention, concentration, language, learning, semantic processing, orientation, executive function, and aspects of emotional regulation. Cognitive impairment exists on a spectrum that ranges from mild cognitive impairment (MCI) to dementia, with deficits across multiple domains such as episodic memory, executive function, attention, processing speed, language fluency, and working memory [5]. In contrast to dementia, individuals with MCI generally retain the ability to perform daily activities independently despite measurable cognitive decline [6].

Accumulating epidemiological evidence has consistently demonstrated an association between hearing loss and cognitive impairment [7,8]. Consequently, age-related hearing loss is increasingly regarded as a potentially modifiable risk factor for cognitive decline and dementia. Notably, the 2020 Lancet Commission on Dementia Prevention, Intervention and Care identified midlife hearing loss as the single largest potentially modifiable risk factor for dementia, a conclusion reaffirmed in the 2024 update of the Commission [9,10].

Several recent reviews have synthesized the available evidence, but several gaps remain. Specifically, most have focused predominantly on age-related hearing loss and older adults [7,11,12,13], with comparatively less attention given to integrating evidence across the lifespan, including the distinct relationship between hearing impairment and cognition during childhood. Others have focused on the intervention side (hearing aids and cochlear implants) [14,15]. According to Ying et al., the generally low methodological quality and the limited certainty of evidence in the available systematic reviews and meta-analyses on the issue constitute a major limitation [16].

Furthermore, the cognitive impact of hearing loss in young and middle-aged adults was not examined systematically until very recently [17]. The link between childhood hearing loss and cognitive impairment, to the best of our knowledge, is not covered in a dedicated review but rather in reviews that include pediatric populations with hearing aids and cochlear implants [18,19]. In addition, most reviews pool studies that are heterogeneous in terms of study design, audiometric definitions of hearing loss, and incompatible effect measures [16]. Finally, the search dates of previous systematic reviews and meta-analyses, such as those by Loughrey et al. [7] and Yeo et al. [14], preceded more recent adequately powered randomized trials of hearing interventions and large cohorts [20,21,22,23,24,25].

Against this background, the present review aims to provide a comprehensive and critical overview of the relationship between hearing loss and cognition across the lifespan. It synthesizes epidemiological evidence, proposed biological mechanisms, and the effects of auditory rehabilitation—including hearing aids and cochlear implants—while extending the scope to encompass pediatric populations.

Historically, research on hearing loss and cognition has focused overwhelmingly on older adults, yet the relationship between auditory experience and cognitive function is not confined to later life. It begins in infancy, when auditory input shapes the developing brain, and continues through young adulthood and into old age, when an already-established cognitive system must be maintained in the face of progressive sensory decline. This review therefore adopts a lifespan perspective and is organized chronologically. We first consider hearing and cognitive development in childhood, where the central issue is prelingual auditory deprivation during sensitive periods of brain maturation. We then turn to young and middle-aged adults, in whom early hearing loss may act as an emerging risk factor, before examining older adults, in whom age-related hearing loss is linked to accelerated cognitive decline and dementia. Having described these three populations, we set out an explicit justification for considering them together despite their differences. Only after these adult findings are set against the developmental evidence from childhood can the hearing–cognition relationship be treated as a continuous, lifelong phenomenon rather than a set of disconnected, age-specific observations.

Moreover, we aim to bring the evidence up to date by incorporating the most recent data that postdate previous systematic reviews and meta-analyses. The epidemiological association, the proposed biological mechanisms, and the effects of both hearing aids and cochlear implants are integrated within a single narrative.

## 2. Materials and Methods

This narrative review was based on a comprehensive search of the peer-reviewed literature. Electronic searches were conducted across the PubMed, Scopus, and Web of Science databases, using combinations of the following keywords: (“hearing loss”), (presbycusis), (“age-related hearing loss”), (“cognitive decline”), (“cognitive impairment”), (“mild cognitive impairment”), (dementia), (“hearing aids”), (“cochlear implants”), (cognition), (children), (“pediatric hearing loss”), (“middle-aged adults”), (“young adults”), and (adulthood). The literature search included articles published up to August 2026.

Articles were eligible for inclusion if they were published in English, French, Italian or German and consisted of original research articles, systematic or narrative reviews, meta-analyses, or clinical trials addressing the association between hearing loss and cognitive decline, the potential mechanisms linking these conditions, or the effects of auditory rehabilitation on cognitive outcomes. Studies involving both adult and pediatric populations were considered, while conference abstracts and editorials were excluded.

Potentially relevant publications were assessed in full text by three authors, and the reference lists of selected articles were manually searched to identify additional relevant studies. Given the narrative nature of this review, studies were selected on the basis of their scientific relevance, methodological quality, and contribution to the current understanding of the relationship between hearing loss and cognition, with particular emphasis given to high-quality systematic reviews, meta-analyses, randomized controlled trials, and large prospective cohort studies.

## 3. Hearing and Cognitive Development in Childhood

The lifespan account begins in childhood, the earliest and in some respects most consequential point on the hearing–cognition trajectory, where the question is not how cognitive ability is preserved but how it is first built.

Although most research on hearing loss and cognition concerns older adults, hearing loss also affects cognition in childhood, albeit through a different mechanism. In older adults, hearing loss is largely considered a modifiable risk factor for cognitive decline, whereas in children, the central concern is the impact of auditory deprivation on cognitive development and executive function during a period of experience-dependent neural maturation [26]. This distinction is clinically important, as the pediatric goal is not to slow decline but to establish the sensory and linguistic foundations on which higher-order cognition is built. Hearing loss in children is common, affecting nearly 1 in 5 children by 18 years of age, and without timely rehabilitation, it can compromise speech, language, educational, cognitive, and psychosocial development [27].

Prelingually deaf children who do not receive auditory intervention may communicate through spoken or signed language. However, reliance on spoken language alone often fails to reach a high communicative standard because of heavy dependence on lip-reading, whereas sign language is considered functionally equivalent to spoken language. Evidence accumulated over decades indicates that language modality does not, in itself, impair general intellectual development; rather, cognitive consequences arise principally through the deprivation of experience and stimulation [26]. The critical factor, therefore, is access to rich and perceptible language during the early childhood years, as the neurocritical period for learning a first language spans roughly from birth to age 5. Children who become deaf or hard of hearing during this period are at risk of language deprivation and subsequent language difficulties, as well as impairments in higher cognitive functions such as sequential memory, cause-and-effect reasoning, and mood regulation. Ensuring full access to language, either spoken or via hearing/visual technology, or both, can prevent these permanent sequelae [28].

Children with hearing impairment characteristically experience difficulty in spoken-language development. The central auditory system, and the central nervous system more broadly, retains a high capacity for neuroplastic change during early childhood, with a sensitive period for central auditory development in the first few years of life [29,30]. Cortical development accelerates rapidly during the first years of life. This period of high brain plasticity supports experience-driven learning, especially the acquisition of spoken-language skills [31]. Hearing deprivation during this window prevents functional maturation and delays auditory object formation [31]. Neuroimaging supports the view that the first years of life are a sensitive period for brain development. In children with hearing loss, early access to sound helps preserve normal brain connectivity, while delayed access can lead to changes in both auditory and other brain networks [32].

For this reason, intervention is recommended as early as possible—ideally before 12 months of age. Management typically begins with hearing aids, while cochlear implantation is generally considered for children with bilateral or, more recently, unilateral, sensorineural hearing loss exceeding approximately 80 dB HL after a trial period of hearing-aid use and rehabilitation [30]. Effective stimulation through a cochlear implant during the sensitive period can exploit this plasticity and promote auditory maturation, whereas late intervention leads to insufficient adaptation and poorer outcomes [31]. Consistent with this, data show that language benefits from early intervention grow with the degree of loss. Specifically, children whose hearing is amplified at 3 months outperform those whose hearing is amplified at 24 months, and children implanted at 6 months outperform those implanted at 24 months by more than 20 language score points [33].

The human nervous system can be conceptualized as an information-processing hierarchy that progresses from sensation to perception, attention, learning, and memory, with the capacity to encode and store both verbal and visuospatial information in working memory in support of language and academic achievement. Deaf or hard-of-hearing children who lack auditory input during this developmental window may show deficient maturation of this system, and a paucity of verbal information can delay language acquisition.

Even after cochlear implantation, children tend to perform below normal-hearing peers on many cognitive tasks. Studies have identified domain-specific deficits in verbal working memory—linked to degraded processing of verbal information—whereas visuospatial working memory is often comparable to that of normal-hearing children. When directly comparing 5- to 9-year-old children, those with cochlear implants fared significantly worse than normal-hearing peers in verbal working memory, receptive vocabulary, and verbally mediated reasoning, even when controlling for age and maternal education. However, there was no difference in visuospatial working memory and matrix reasoning skills that require minimal verbal labeling [34]. A dissociation is observed in school-aged subjects, showing low ability to store and process verbal but not nonverbal information, suggesting that implant users may rely on nonverbal strategies [35]. These verbal-domain deficits have been attributed in part to the spectral degradation of the auditory signal delivered by the cochlear implant [34,36]. According to research, people with cochlear implants usually exhibit increased phonological confusion in their verbal memory, probably because their phonological representations are less precise. These difficulties are related to delays in spoken language [37]. However, the magnitude of these deficits is modifiable; outcomes are better with earlier implantation and bilateral implants, which can improve language, working memory, and attention [38].

Beyond verbal working memory, childhood hearing loss may affect broader executive functioning. It has also been shown that long-term cochlear implant users show poorer executive functioning than normal-hearing children, including deficits in working memory, processing speed, and inhibitory control, suggesting that reduced auditory stimulation may influence wider neurocognitive development [39,40]. Executive functioning includes various skills such as working memory, controlled attention, self-monitoring, organization and cognitive flexibility. Children with cochlear implants may have difficulties in these areas and more specifically in verbal working memory, controlled cognitive fluency, attention, and concentration [31]. In a prospective study, children with implants had 2–5 times greater risk of clinically significant executive function deficits than normal-hearing peers across most domains, including comprehension and learning, factual memory, attention, sequential processing, working memory, and problem-solving. In contrast, visual–spatial skills were less affected [41]. Importantly, these executive function differences can be explained by several factors, including reduced or delayed access to sound, early auditory deprivation and differences in language experience. Executive functioning and language development also seem to influence each other. Better access to sound and consistent device use are linked to better vocabulary and verbal working memory in children with hearing loss [42]. These findings support the need for early screening and intervention for executive function difficulties [41,43].

Cognitive status is itself an important determinant of speech-perception outcomes after cochlear implantation, which makes the early identification of cognitive difficulties valuable; reported outcomes nonetheless vary across studies, with some showing normal rates of behavioral adaptation in cognitively impaired children and others showing poorer trajectories [18]. Notably, developmental delay does not necessarily mean that a child cannot derive benefit from a cochlear implant. Children who exhibit developmental problems in their early stages may continue to develop in various areas such as cognition, adaptation, language, and auditory skills following a cochlear implant [44]. A child’s early language skills are among the best predictors of later language and cognitive abilities, including memory, and the link between verbal working memory and language appears stronger in cochlear implant users than in normal-hearing children [40].

A lack of auditory stimulation is thought to provoke atypical development of cognitive functions, and the resumption of auditory input after cochlear implantation may re-engage neuroplastic mechanisms and support cognitive improvement. Cognitive status in children should therefore be regarded both as a co-factor that influences speech-perception outcomes and as a function that hearing loss itself may partially impair [18].

In conclusion, early auditory deprivation and delayed language development may have lasting effects on later cognitive and auditory function. Early auditory stimulation and timely intervention with hearing devices are therefore essential to support cognitive development and to prevent later difficulties in children with hearing loss. The best outcomes will therefore probably be obtained if early detection through screening at birth, early amplification or implantation, language habilitation, and monitoring for working memory and executive function difficulties are undertaken to address those problems while neuroplasticity remains high [33,41,43,45].

## 4. Hearing and Cognitive Decline in Young and Middle-Aged Adults

Between the developmental concerns of childhood and the better-documented decline of old age lies a less studied group: young and middle-aged adults. In this population, the auditory and cognitive systems are fully developed, yet hearing loss acquired in early or midlife may represent one of the earliest modifiable contributors to a cognitive trajectory that becomes clinically apparent only decades later.

Hearing difficulties are also relevant to young adults, as they may result from noise exposure, ototoxic drugs, and even genetic and congenital factors. It is important to become familiar with this connection early in life, since the relationship between cognitive and auditory function appears to be a continuum rather than something limited to old age and childhood [27,46,47].

Hearing loss throughout adulthood has been associated with poor cognitive performance and an increased risk of cognitive decline. Specifically, a meta-analysis of several cohort studies showed that hearing loss in adults was associated with a higher risk of dementia, mild cognitive impairment, and Alzheimer’s disease, with a dose–response relationship observed such that each 10-decibel worsening in hearing was associated with a 16% increase in the risk of dementia [48].

It is important to note that the Maastricht Aging Study, which included participants aged 24 to 82, showed hearing loss, either already present or developed later, to be linked to a faster rate of cognitive decline, particularly in verbal memory, processing speed, and executive function. In addition, the onset of hearing loss was found to precede cognitive decline [20]. Moreover, subclinical hearing loss, that is, hearing loss falling within the range traditionally regarded as normal, has been associated with poorer cognitive function [49].

Several mechanisms have been proposed to explain the connection between hearing loss and cognitive decline in pediatric and older populations, and these could apply to young adults as well. The first is the cognitive load hypothesis, which holds that the effort involved in listening depletes the cognitive resources needed for other tasks, such as memory encoding and executive function [50]. The second is the sensory deprivation hypothesis, which proposes that reduced auditory stimulation may contribute to structural brain changes, including atrophy of the auditory cortex and other brain regions.

Ultimately, hearing loss may contribute to social isolation, a factor that itself increases the risk of cognitive impairment, as difficulties in speech understanding can reduce social interaction [51,52].

Because these mechanisms could potentially be modified, early detection and intervention are important. The use of hearing aids and cochlear implants is associated with an estimated 19% reduction in the risk of long-term cognitive decline, along with some short-term improvement in cognitive test scores, and individuals whose hearing loss is treated show better cognitive outcomes than those whose hearing loss remains untreated [14,53]. A recent meta-analysis by Satheesan et al. (2025) found that untreated hearing loss in young and middle-aged adults was associated with significantly poorer overall memory, executive function, and global cognitive status [17].

Although the evidence is based mainly on observational studies and notably lacks randomized controlled trials in younger and middle-aged adults, it nonetheless points to the need for timely hearing tests, hearing conservation from both occupational and leisure noise exposure, and prompt rehabilitation.

In sum, adult hearing loss is a potentially modifiable risk factor for impaired cognitive function and accelerated cognitive decline, likely resulting from greater cognitive effort, sensory deprivation leading to changes in the central nervous system, and social isolation. Maintaining good hearing health during young adulthood may therefore be advantageous for long-term cognitive function, though further research is needed.

## 5. Hearing Loss and Cognitive Decline in Older Adults and the Cognitive Domains Affected

The relationship between presbycusis and cognitive decline has been extensively investigated over the past decade. The available evidence supporting this association is summarized below.

An influential study by Lin et al. in 2011 showed that the risk of dementia increases as hearing thresholds worsen, following a log-linear relationship [54]. The authors also reported that more than one-third of dementia cases in people older than 60 years were attributable to hearing impairment and that all subtypes of dementia were associated with hearing loss. According to this study, all of the dominant theories explaining the link between presbycusis and cognitive decline—except the overdiagnosis hypothesis—could be valid; the overdiagnosis explanation was made less likely by excluding individuals with documented cognitive impairment at baseline. The authors further proposed that hearing impairment might directly influence the pathology of Alzheimer’s disease. Self-reported hearing-aid use was not found to reduce the incidence of dementia, although this particular finding was not robustly supported [54].

In a subsequent study, Lin et al. (2013) reported a higher risk (24%) and an accelerated rate (30–40%) of cognitive deterioration among hearing-impaired older adults compared with normal-hearing individuals [55]. No statistically significant reduction in the incidence of cognitive impairment was demonstrated with hearing-aid use. Male sex, older age, White race, and smoking were identified as factors predisposing individuals to hearing loss [55].

The overall association has subsequently been reinforced by higher-level evidence. A meta-analysis by Loughrey et al. confirmed that age-related hearing loss was associated with cognitive decline and dementia, although not specifically with Alzheimer’s disease or vascular dementia. Vascular disease and impaired verbal communication in hearing-impaired individuals were proposed as possible contributors to this decline [7].

Beyond the overall association between hearing loss and dementia, age-related hearing loss has been linked to impairment across specific cognitive domains. Untreated hearing loss in older adults has been associated with poorer executive function, processing speed, episodic memory, and language fluency relative to normal-hearing peers, while executive dysfunction in particular appears to increase the risk of subsequent dementia [5].

Supporting these findings, Soons et al. (2024) demonstrated in a 12-year prospective cohort study that both prevalent and incident hearing loss were associated with accelerated decline in verbal memory, information-processing speed, and executive function [20]. Importantly, hearing loss preceded the onset of cognitive decline, supporting the hypothesis that hearing impairment may be an independent risk factor for cognitive deterioration rather than merely a coexisting manifestation of aging [20].

Recent Mendelian randomization evidence further supports a potential causal relationship between hearing impairment and cognitive decline, suggesting that the two do not simply coexist but that hearing loss may actively contribute to cognitive deterioration. Loneliness, depressive symptoms, and cortical atrophy—particularly involving the middle temporal gyrus—have been proposed as potential mediators of this association. These findings reinforce the view that early detection and management of hearing loss may represent a promising avenue for dementia prevention [56].

Taken together, the available evidence consistently supports an association between hearing loss and cognitive decline across multiple cognitive domains and age groups. However, most evidence remains observational, precluding definitive conclusions regarding causality.

Furthermore, an important methodological consideration when interpreting studies on hearing loss and cognition is the potential influence of hearing impairment on cognitive test performance itself. Impaired cognitive test results may partly reflect a reduced ability to hear and follow test instructions rather than true neurodegenerative decline, a caveat that complicates interpretation across this literature [5].

Commonly used cognitive screening instruments, including the Mini-Mental State Examination (MMSE) and the Montreal Cognitive Assessment (MoCA), contain spoken instructions and auditory-dependent test items [57,58]. Consequently, reduced audibility may adversely affect cognitive test performance independently of underlying cognitive function, potentially leading to an overestimation of cognitive impairment and inaccurate cognitive classification [57,58,59].

To reduce this auditory bias, hearing-adapted cognitive screening instruments, such as the Hearing-Impaired Montreal Cognitive Assessment (HI-MoCA) [59], have been developed using different approaches, including written presentation of test instructions and stimuli or the removal of auditory-dependent test items [57]. However, despite these efforts, only a limited number of hearing-adapted instruments are currently available, and the validity of existing ones remains to be fully established [58]. Moreover, most available versions lack robust normative data, while adaptations—especially those involving item deletion—may adversely affect the psychometric properties of the original instruments and potentially reduce diagnostic sensitivity and specificity [57,59].

Notably, in the study by Dupuis et al. (2015), excluding hearing-dependent MoCA items reduced, but did not eliminate, the performance difference between individuals with and without hearing loss, indicating that sensory-related test demands alone do not fully account for the observed differences in cognitive performance [60].

Collectively, these findings emphasize that cognitive assessment in individuals with hearing loss should account for hearing status, as well as the current limitations of both conventional and hearing-adapted cognitive screening instruments.

## 6. Reconciling the Three Populations: Prelingual Children, Young Adults, and Older Adults

The three populations considered above—prelingually hearing-impaired children, young and middle-aged adults, and older adults with age-related hearing loss—differ in fundamental ways, and it is reasonable to ask whether they can meaningfully be discussed within a single framework. In prelingual hearing loss, auditory deprivation occurs before language is acquired, during sensitive periods of cortical development; its cognitive consequences are therefore developmental, affecting the acquisition of spoken language, verbal working memory, and executive function [28,31]. Early cochlear implantation seeks to establish a developmental trajectory that would otherwise fail to form [44].

In postlingual and age-related hearing loss, by contrast, an already-mature cognitive system is exposed to progressive sensory decline. The consequences reflect processes of maintenance and loss, such as increased listening effort, reduced afferent stimulation, and social isolation, and intervention seeks to preserve function rather than to build it [61]. Young and middle-aged adults occupy an intermediate position, in which a fully developed system first begins to sustain the effects of sensory deprivation [50,62].

We therefore do not treat these populations as directly comparable. Their mechanisms, outcome measures, time courses, and the reversibility of their deficits differ, and it would be inappropriate to pool their findings or to compare effect sizes across them. Rather, we consider them together because, taken as a whole, they reveal a single unifying principle: that access to sound is tied to cognitive function throughout life, and that restoring auditory input, whether to build cognition in a developing child or to preserve it in an aging adult, can favorably influence the cognitive trajectory. Viewed through this lens, the developmental stage at which hearing loss occurs is not a confounder to be explained away but the very variable that determines whether the clinical priority is the construction or the preservation of cognitive ability. It is this continuity across the entire lifespan that motivates the integrated approach taken in this review.

## 7. Mechanisms Linking Hearing Loss and Cognitive Decline

A model proposed by the European auditory processing disorder (APD) group and the EFAS APD Working Group describes bidirectional relationships among hearing sensitivity, auditory processing, cognition, language, and thought processes [63]. Several mechanisms have been advanced to explain how age-related hearing loss may contribute to cognitive deterioration, and these are most often grouped into four overlapping theoretical models [61].

### 7.1. Theoretical Models Relating Presbycusis to Cognition

The first is the cognitive load (or “information degradation”) hypothesis, which holds that every task requires a finite quantity of cognitive resources. When the afferent auditory signal is degraded, individuals must allocate additional listening effort to comprehend speech, leaving fewer resources available for other operations such as encoding into working memory. Over time, this sustained reallocation—together with maladaptive neuroplastic reorganization of higher-order cortical regions—is thought to compromise functions such as working memory. Under this model, hearing aids should reduce listening effort and free cognitive resources, potentially slowing brain atrophy and cognitive decline [61].

The second is the common cause hypothesis, which posits that shared neuropathological factors drive both hearing loss and cognitive decline. Impaired microcirculation is considered a major candidate, since atherosclerosis, smoking, and diabetes are all linked to both peripheral hearing loss and neurodegeneration; oxidative stress and genetic factors (e.g., APOE genotype) may also contribute. Under this model, hearing aids would not be expected to alter the cognitive trajectory, because the underlying driver affects the two systems through independent pathways [61].

The third is the cascade hypothesis, grounded in experience-dependent neuroplasticity. Peripheral hearing loss is thought to (i) reduce activity in the central auditory system, leading to neurodegeneration of the corresponding cortical areas, and (ii) restrict verbal communication, contributing to social isolation and depression—both of which impair cognition. In this model, hearing aids could restore auditory activity and verbal communication, thereby addressing two causative pathways [61].

The fourth is the overdiagnosis, or “harbinger” hypothesis, which encompasses two possibilities: that poor neuropsychological test performance in people who cannot hear instructions leads to a false diagnosis of cognitive impairment (and, conversely, that hearing loss may be misdiagnosed in people with dementia) and that hearing loss is an early marker of incipient dementia. In the latter case, hearing intervention would chiefly help to avoid misclassification, although whether it can delay the onset of dementia remains uncertain [61]. The four models, and the contrasting predictions they make about the value of amplification, are summarized in Table 1.

### 7.2. Neurobiological Changes Associated with Hearing Loss

These theoretical models are supported by evidence of functional and structural degeneration of both the peripheral and central auditory systems as presbycusis progresses. Whether such changes drive cognitive decline or instead reflect a generalized process of unknown cause remains unresolved. Because the auditory, visual, motor, and cognitive cortical domains are interconnected, alterations in the auditory system may be mirrored by changes elsewhere [61,64].

Reported structural changes associated with hearing loss include reduced gray matter volume in the auditory and prefrontal cortices bilaterally and decreased white matter volume in the auditory radiation, as well as a relocalization of auditory processing from temporal to frontal cortical areas [64,65]. Belkhiria et al. (2019) reported that presbycusis with cochlear-amplifier dysfunction was accompanied by atrophy of the cingulate cortex and parahippocampus, with cingulate atrophy associated with deficits in episodic and working memory, language, and visuoconstructive ability [66].

More recent large, multi-cohort analyses have linked hearing impairment to lower volume of the temporal cortex, hippocampus, and related regions, reduced white matter integrity, and increased markers of tau pathology, with brain atrophy and tau partly mediating the association between hearing impairment and cognitive decline [62]. Given the central role of the hippocampus in both memory and auditory processing, these findings provide additional neurobiological support for the link between hearing loss and cognitive decline.

It is likely that all four theories apply, to varying degrees, in any given individual with coexisting hearing loss and cognitive impairment [61]. Consistent with this view, current evidence indicates that no single factor fully explains the association; rather, multiple interacting factors—sensory, cognitive, vascular, genetic, and social—appear to be involved, underscoring the complexity of the relationship [67].

## 8. The Effect of Hearing Aids on Cognition in Adults

If hearing loss contributes to cognitive decline through reduced auditory stimulation or impaired communication, auditory rehabilitation could, in principle, modify this process. Hearing aids remain the first-line intervention for most older adults with presbycusis, and a substantial body of observational and interventional research has examined their effect on cognition [68].

Cross-sectional data indicate that individuals with treated hearing loss show cognitive performance closer to that of normal-hearing individuals than those with untreated loss do, suggesting that rehabilitation may help preserve cognition [5].

In an 18-year national study in the USA, hearing-aid use was associated with a slower decline in episodic memory among adults aged 50 years and older; the authors interpreted this pattern as more consistent with the cascade hypothesis than with the common cause hypothesis [69]. In a randomized pilot trial, Deal et al. (2017) studied adults with hearing loss and without dementia and found better psychosocial and cognitive outcomes at six months in the group fitted with hearing aids than in a health-education control group [70].

The landmark ACHIEVE randomized controlled trial subsequently provided higher-quality evidence on the effect of a hearing intervention (hearing aids plus rehabilitative support) on cognition. Although the intervention did not significantly slow cognitive decline in the overall study population, a prespecified subgroup of older adults at higher risk of decline—older participants with more vascular risk factors, recruited from the ARIC cohort—experienced an approximately 48% reduction in the rate of cognitive decline over three years [21]. A subsequent secondary analysis confirmed that the cognitive benefit of hearing intervention varied according to baseline risk, being concentrated among those most vulnerable to decline [22]. These findings suggest that the cognitive benefits of hearing rehabilitation may not be universal but may be most relevant to older adults at elevated risk.

Building on these findings, a large pooled longitudinal analysis including more than 61,000 older adults from seven international cohorts demonstrated that the cognitive benefits associated with hearing-aid use depend on the effectiveness of hearing rehabilitation. Specifically, a lower risk of probable dementia was observed only among hearing-aid users who reported meaningful hearing improvement, whereas those reporting limited benefit had a dementia risk comparable to that of non-users. This highlights that these cognitive benefits are likely contingent on successful hearing rehabilitation and may be underestimated when hearing-aid effectiveness is not taken into account [71].

A large population-based cohort study further showed that the association between hearing-aid use and cognitive outcomes is modified by hearing loss severity. Hearing-aid users with mild hearing loss exhibited cognitive performance comparable to that of normal-hearing individuals, suggesting that early hearing-aid intervention may mitigate the cognitive burden associated with hearing loss. In contrast, individuals with moderate-to-severe hearing loss continued to exhibit poorer cognitive performance than normal-hearing participants regardless of hearing-aid use, indicating that the cognitive benefits of hearing-aid use may be attenuated in more advanced hearing loss. A plausible explanation is that, in more advanced hearing loss, amplification cannot fully compensate for cochlear degeneration, leading to incomplete restoration of auditory input and thereby diminishing the potential cognitive benefits of hearing-aid use [72]. Likewise, meta-analytic evidence largely supports a beneficial effect of hearing aids. A recent meta-analysis by Liu et al. (2026), incorporating a very large pooled sample, found that hearing-aid use was associated with improved global cognition, memory, and executive function and with a reduced risk of cognitive impairment in older adults with hearing loss [73]. An earlier meta-analysis similarly reported improvements in global cognition, executive function, and episodic memory among hearing-aid users without dementia, although no clear benefit was observed in those with established Alzheimer’s disease [74].

Large cohort and pooled analyses reinforce these conclusions. A Danish population-based cohort of more than 570,000 adults aged over 50 years found that hearing-aid users had a significantly lower risk of incident dementia than individuals with untreated hearing loss [23]. A systematic review and meta-analysis by Yeo et al. (2023) likewise concluded that the use of hearing-restorative devices was associated with a lower hazard of cognitive decline and dementia [14].

Not all evidence is unequivocal, however. The relationship between hearing-aid use and cognition is partly bidirectional—better cognition facilitates successful device uptake and continued use—so observational associations may be inflated by reverse causation and by the characteristics of people who choose to wear hearing aids [75]. In a meta-analysis by Taljaard et al. (2016), hearing loss was associated with poorer cognition regardless of treatment status, but the use of hearing aids (or cochlear implants) was associated with better cognitive outcomes than no treatment; many of the included studies, nonetheless, lacked randomization [53].

Although positive cognitive outcomes have been reported in people with normal cognition who use hearing devices, far less research has addressed older adults who already have dementia. In studies of hearing aids or assistive listening devices in this population, results have been variable, although symptoms such as verbal agitation and depression were generally ameliorated [76]. Overall, hearing aids appear beneficial for older adults with hearing loss and intact cognition and may relieve some dementia-related symptoms in those with established cognitive impairment, but the evidence in the latter group is of lower quality and warrants further investigation [76].

## 9. The Effect of Cochlear Implants and Other Auditory Interventions on Cognition in Adults

An increasing number of older adults with severe-to-profound hearing loss, or with poor speech discrimination that is inadequately improved by hearing aids, now receive cochlear implants. Because cochlear implantation restores access to sound through direct electrical stimulation of the auditory nerve, it offers a distinct opportunity to study the effect of auditory restoration on cognition [77].

A systematic review by Miller et al. (2015) of adults aged 65 years and older with sensorineural hearing loss treated with cochlear implants concluded that implantation improved speech perception and overall quality of life, although the authors emphasized that further, higher-quality research was needed [77]. Mosnier et al. (2015) reported that cognitive performance improved in a majority of elderly patients in the year following cochlear implantation, with gains accompanied by improvements in mood and quality of life [78]. Cognitive function may also be a more reliable predictor of postoperative speech-recognition improvement than chronological age [79].

Castiglione et al. (2016) studied adults aged 65 years and older using hearing aids or cochlear implants [80]. Short-term memory improved in hearing-aid users, whereas cochlear implant recipients showed cognitive and depression scores comparable to those of normal-hearing peers, together with improvements in long-term memory and in visuospatial and logical-executive skills [80].

Additional evidence for the long-term cognitive effects of cochlear implantation was provided by Völter et al., who followed older adults with severe postlingual hearing loss for up to 65 months after implantation. Significant improvements were observed across several cognitive domains, including attention, working memory, delayed recall, verbal fluency, and inhibition. These improvements emerged predominantly during the first postoperative year and subsequently remained stable throughout follow-up, whereas individuals with poorer preoperative cognitive performance appeared to derive the greatest benefit from implantation [81].

Further insight into the long-term cognitive effects of cochlear implantation comes from a 4.5-year prospective longitudinal study of older adults with severe-to-profound hearing loss, in which cochlear implant recipients demonstrated significant improvements in executive function and working memory while maintaining stable performance in attention, psychomotor function, and visual learning. These findings suggest that cochlear implantation may help preserve cognitive function, delay cognitive decline, and improve cognitive performance in this population [82].

Völter et al. further refined the understanding of long-term cognitive trajectories after cochlear implantation by applying nonlinear growth modeling over approximately five years of follow-up. Improvements were primarily observed in immediate and delayed recall, with the greatest gains occurring during the first postoperative year; thereafter, cognitive performance largely stabilized rather than continuing to improve. This pattern suggests an early enhancement of memory function (a “booster effect”), followed by sustained maintenance of these benefits, in contrast to the progressive cognitive decline typically observed in untreated hearing loss. The authors also emphasized that, despite these encouraging findings, current evidence remains insufficient to conclude that cochlear implantation alone prevents age-related cognitive decline or dementia [83].

The same group has since extended this cohort to nearly nine years and addressed the question of how far such trajectories depend on the statistical model chosen. Seventy-five recipients aged 50 years or older were assessed on attention, immediate and delayed recall, working memory and verbal fluency before implantation and at 12, 24, 54 and 99 months afterwards. Three linear mixed-effects specifications were fitted to the same data. All three converged on the same core result of significant improvement across all five cognitive outcomes at 12 and 24 months. Beyond two years, however, the trajectories diverged by domain rather than following a single common curve, which suggests that the cognitive benefit of implantation is not uniform across cognitive functions. Thus, apparent discrepancies between published long-term studies may partly reflect differing modeling assumptions rather than differing patient outcomes [84,85].

A recurring limitation of this literature is the absence of a control group, since withholding implantation from severely hearing-impaired candidates for several years is not ethically defensible. One recent study addressed this by comparing verbal fluency trajectories in 74 cochlear implant recipients with those of 383 participants with untreated hearing loss drawn from the English Longitudinal Study of Ageing, over a comparable period of up to nine years. The untreated group declined steadily, whereas the implanted group followed an inverted-U trajectory of initial improvement followed by a plateau over the standardized follow-up that persisted after propensity score matching for age, sex and education [85]. Verbal fluency is a useful outcome in this context because it draws on semantic retrieval, executive control and processing speed while depending relatively little on peripheral hearing, and because decline in fluency is itself predictive of dementia risk. The design nonetheless remains observational; the two cohorts used different fluency tasks, and the implanted samples in these reports overlap substantially with those in the earlier analyses cited above [81,83].

A systematic review by Carasek et al. (2022) likewise found that both cochlear implants and hearing aids were associated with cognitive benefit in older adults [15], and the meta-analysis by Yeo et al. (2023) included cochlear implant studies in reaching its overall conclusion that hearing restoration is associated with reduced cognitive decline [14].

A recent narrative review similarly reported cognitive benefits during the first postoperative year, while emphasizing the lack of standardized assessment protocols and long-term controlled studies [86,87]. Consistent with the domain-specific pattern described above, a prospective study of older recipients followed for four years with the Repeatable Battery for the Assessment of Neuropsychological Status for Hearing-Impaired Individuals (RBANS-H) found that the persistence of cognitive improvement varied across domains, with gains in some areas maintained while others returned towards baseline [85]. Taken together, these longer series suggest that the early post-implantation gain is robust and reproducible, whereas its maintenance beyond two years is domain-dependent and, at present, inconsistently reported.

Beyond devices, other auditory interventions—including structured auditory training and combined audiological rehabilitation programs—are sometimes used alongside amplification and may contribute to the cognitive and communicative benefits observed with rehabilitation, although the independent cognitive effect of such training remains poorly defined [80].

Overall, the evidence suggests that cochlear implantation, like hearing-aid use, is associated with improvements in speech perception, quality of life, and several cognitive domains in older adults. However, the cochlear implant literature is dominated by small, non-randomized studies, and robust trials are still required before firm conclusions can be drawn about a causal cognitive benefit [14,15].

## 10. Conclusions

Hearing loss and cognitive decline are closely intertwined across the life course. In older adults, a consistent body of epidemiological evidence links age-related hearing loss to accelerated cognitive decline and to an increased risk of dementia, affecting domains that include episodic memory, executive function, processing speed, and language fluency. Several complementary mechanisms—captured by the cognitive load, common cause, cascade, and overdiagnosis hypotheses and supported by neuroimaging evidence of cortical and hippocampal atrophy—probably operate together rather than in isolation, which helps to explain why no single model fully accounts for the association.

Read across the lifespan, these studies tell a single, coherent story. In childhood, hearing loss threatens the very construction of cognitive and linguistic ability, and timely device fitting within sensitive developmental periods is critical. In young and middle-aged adults, it may represent one of the earliest modifiable contributions to a cognitive trajectory that manifests only much later. In older adults, it accelerates decline in an already-mature system. Although the mechanisms and the goals of intervention differ at each stage—building cognition early in life, preserving it later—the through-line is consistent: auditory input matters for cognition from infancy to old age, and restoring it can help.

The therapeutic implications are encouraging but require careful interpretation. Evidence from observational studies, large prospective cohorts, longitudinal investigations, and meta-analyses consistently associates hearing-aid and cochlear implant use with more favorable cognitive outcomes. The ACHIEVE trial demonstrated that hearing intervention can meaningfully slow cognitive decline in older adults at elevated risk. Emerging evidence further indicates that the cognitive benefits associated with hearing-aid use may be influenced by the effectiveness of auditory rehabilitation and the severity of hearing loss. For cochlear implantation, recent longitudinal studies have strengthened the evidence for favorable long-term cognitive trajectories, characterized by sustained cognitive performance over time. Nevertheless, evidence in individuals with established dementia remains limited; high-quality randomized studies of cochlear implantation are still lacking, and reverse causation continues to complicate the interpretation of observational findings.

In children, the central issue is developmental: early auditory deprivation can influence the maturation of verbal working memory and executive function, underscoring the importance of early identification and timely auditory intervention to optimize both cognitive and communicative development.

Overall, hearing loss is best regarded as an important and potentially modifiable contributor to cognitive decline across the lifespan, rather than a proven determinant of dementia. Early detection and appropriate auditory rehabilitation represent low-risk, potentially high-value strategies for protecting cognitive health, particularly in high-risk older adults and in young children. Future adequately powered, long-term randomized controlled trials—especially in cochlear implant recipients and individuals with pre-existing cognitive impairment—are needed to clarify which patients derive the greatest cognitive benefit and to determine the optimal timing of intervention.

## Figures and Tables

**Table 1:** Principal theoretical models linking hearing loss to cognitive decline and their predictions regarding hearing-aid use.

Hypothesis	Core Proposed Mechanism	Predicted Effect of Hearing Aids
Cognitive load (information degradation)	Degraded auditory input increases listening effort, depleting shared cognitive resources and driving maladaptive cortical reorganization.	Benefit expected: reduced effort frees cognitive resources.
Common cause	Shared pathology (e.g., microvascular disease, oxidative stress, and genetic factors such as APOE) independently damages auditory and cognitive systems.	No benefit expected: the driver is generalized.
Cascade	Reduced auditory input causes central auditory degeneration and, through communication difficulty, social isolation and depression.	Benefit expected: restores input and communication.
Overdiagnosis	Hearing loss biases cognitive testing and/or represents an early marker of incipient dementia.	Mainly prevents misclassification; effect on dementia onset uncertain.

## Data Availability

No new data were created or analyzed in this study. Data sharing is not applicable to this article.
